# Effects of Baduanjin exercise on cognitive frailty, oxidative stress, and chronic inflammation in older adults with cognitive frailty: a randomized controlled trial

**DOI:** 10.3389/fpubh.2024.1385542

**Published:** 2024-05-23

**Authors:** Yu Ye, Mingyue Wan, Huiying Lin, Rui Xia, Jianquan He, Pingting Qiu, Guohua Zheng

**Affiliations:** ^1^College of Nursing and Health Management, Shanghai University of Medicine & Health Sciences, Shanghai, China; ^2^Medical School of Chinese PLA, Chinese PLA General Hospital, Beijing, China; ^3^Department of Rehabilitation Medicine, The Second Medical Center, Chinese PLA General Hospital, National Clinical Research Center for Geriatric Diseases, Beijing, China; ^4^College of Rehabilitation Medicine, Fujian University of Traditional Chinese Medicine, Fuzhou, China

**Keywords:** cognitive frailty, Baduanjin, older adults, oxidative stress, chronic inflammation

## Abstract

**Background:**

Oxidative stress and chronic inflammation play an important role in the pathogenesis process of cognitive frailty (CF). Regular Baduanjin exercise could improve cognitive frailty in older adults, but it is unclear whether the effect of Baduanjin exercise on improving CF is mediated by modulating circulating oxidative stress and inflammatory process.

**Method:**

A total of 102 community-dwelling older adults with CF were recruited and randomly allocated into a 24-week Baduanjin exercise training group or no specific exercise intervention control group at an equal rate. Cognitive function and physical frailty index were assessed using the Montreal Cognitive Assessment (MoCA) and the Edmonton Frail Scale (EFS), as well as the oxidative stress and inflammatory cytokines were measured at baseline and after intervention.

**Result:**

After 24 weeks of intervention, the increased MoCA score (2.51 ± 0.32 points, *p* < 0.001) and the decreased EFS scores (1.94 ± 0.20 points, *p* = 0.012) in the Baduanjin group were significantly higher than those in the control group. Serum antioxidant SOD levels were increased by 10.03 ± 4.73 U/mL (*p* < 0.001), and the prooxidative MDA and 8-iso-PGF2α levels were decreased by −1.08 ± 0.80 nmol/mL (*p* = 0.030) and −86.61 ± 15.03 ng/L (*p* < 0.001) in the Baduanjin training group; while inflammatory cytokines IFN-γ, IL-2 and IL-4 levels were increased (1.08 ± 0.33 pg./mL, *p* = 0.034, 2.74 ± 0.75 pg./mL, *p* = 0.04 and 1.48 ± 0.35 pg./mL, *p* = 0.042). In addition, a mediation effect that Baduanjin training improved cognitive ability mediated by an increase of circulating IFN-γ and IL-2 levels were observed in this study.

**Conclusion:**

Regular Baduanjin exercise training could improve the cognitive frailty of the community-dwelling older adults with CF, and modulate oxidative stress and inflammatory processes by reducing circulating pro-oxidative MDA and 8-iso-PGF2α levels and increasing anti-oxidative SOD levels, as well as impacting inflammatory cytokines IFN-γ, IL-2, and IL-4 levels. Nevertheless, the mechanism of Baduanjin exercise mediating oxidative stress and inflammatory processes should be cautious to be explained.

**Clinical trial registration:**

http://www.chictr.org.cn/index.aspx, ChiCTR1800020341.

## Introduction

Cognitive frailty (CF) is defined as a heterogeneous syndrome that involves simultaneously frailty of the physical and cognitive domains, which is more susceptible to adverse health outcomes, such as falls, disability, low quality of life, even if mortality (1–3). In China, the prevalence of CF among community older adults ranges from 1.1–19.86% (4, 5), and is higher in the clinical environment (6, 7).

Although the underlying mechanism of CF remains unclear, oxidative stress and chronic inflammation play an important role in its pathogenesis (8). Increasing pieces of evidence support a link between oxidative damage and frailty or cognitive decline in older people (9). Likewise, researchers have shown that an increased inflammatory condition is positively linked to the pathogenesis of cognitive impairment and physical frailty (10, 11). In the process of aging, the antioxidant defence capacity of older adults is gradually weakened due to the accumulation of the oxidation and peroxidation cellular components under the stimulation of various internal and external factors (12, 13); for example, clinical studies found that higher malondialdehyde (MDA) level and lower superoxide dismutase (SOD) level were associated with muscle atrophy and neurodegeneration (14, 15). While the decline of immune function, the abnormal accumulation of pro-inflammatory factors and the weakening of the anti-inflammatory feedback system directly drive the chronic and low-grade inflammatory response (16); evidence shows that the increased inflammation is also found to have additional predictive effects on the risk of disability in older adults with CF compared to the single physical frailty or cognitive impairment (17).

Many clinical trials have evaluated the effect of exercise intervention on cognitive impairment and physical frailty for older people with different health conditions (1, 18), therefore may be deemed as a promising intervention for improving CF. In addition, there were confirmed that regular exercise intervention with low-moderate intensity could be beneficial to modulate the oxidative stress or systemic low-grade inflammatory reduction of older people (19–23). As one of the most common traditional Chinese Qigong exercises in China, Baduanjin is an aerobic mind–body exercise with low-moderate intensity and emphasizes a combination of physical training with mental focus; interaction between symmetrical physical postures and movements, meditative mind, and breathing techniques in a harmonious manner (24). Due to being less physically and cognitively demanding, Baduanjin exercise is very suitable for older adults. Studies have shown that long-term adherence to Baduanjin can benefit multiple dimensions of health, such as social, psychological, cognitive, and physical functions of middle-aged and older adults (25–27). A previous study reported that a 12-week Baduanjin training could increase antioxidant enzymes and reduce oxidative stress in middle-aged women by modulating the MDA and SOD levels (28). Some studies also reported that the expression of serum IFN-γ, IL-6, TNF-α and other inflammatory factors in older adults people was significantly changed after regular Baduanjin training, suggesting that Baduanjin exercise intervention can alleviate local muscle pain, improve joint mobility, restore neuromuscular function by regulating cytokines (29, 30). Another survey including 156 healthy older adults people confirmed that a 24-week Baduanjin training could reduce the serum MDA, improve SOD activity and total antioxidant capacity (T-AOC), and coordinate the changes of cytokines IL-2, TNF-α and IL-6 in immune function to achieve the anti-aging effect (31). Thus, the improvement of cognition and physiological function of the different populations by regular Baduanjin exercise may be related to regulating the body’s inflammatory condition and oxidative stress level. However, there is no report on the related aspects of CF population. Therefore, it is plausible that the putative benefit of Baduanjin training on CF is more pronounced among older persons. In summary, this study aims to investigate the effect of regular Baduanjin exercise on the CF, inflammation and oxidative stress in community-dweller older adults with CF.

## Methods

### Study design

This study was a two-arm randomized controlled trial with an assessor-blinded, and was conducted between December 2018 and January 2020 in three community centers in Fuzhou city of China, according to the principles of the Declaration of Helsinki. A total of 102 eligible participants were randomly assigned to the Baduanjin exercise training group or the no specific exercise intervention control group with a 1: 1 ratio. The random allocation sequence was generated using the PLAN procedure of the statistical software SAS V.9.0, and was managed by a researcher not involved in the recruitment, intervention and evaluation. The design of the study was detailed in the published protocol (32).

### Participants

Individuals with CF were recruited from the community center of Fuzhou city in China. The inclusion criteria were: age over 60 years old; meeting the CF according to the definition of CF (33); no regular physical exercise for at least half a year (regular exercise means exercise with a frequency of at least twice a week and at least 20 min per session); and the informed consent. Those with uncontrolled hypertension (the systolic blood pressure was greater than 160 mmHg or the diastolic blood pressure was greater than 100 mmHg after taking medicine), severe organ failure, cardiovascular disease, musculoskeletal system diseases and other sports contraindications, and a history of alcohol and drug abuse were excluded. This study was approved by the Ethics Committee of The Second Affiliated Hospital of Fujian University of Traditional Chinese Medicine (No. 2018-KL015) and was registered in China clinical trial registration center with the registration number ChiCTR1800020341.^1^ Informed consent was obtained from all participants involved in the study.

### Interventions

Participants who were allocated to the Baduanjin exercise training group received a 24-week Baduanjin exercise training. The frequency of Baduanjin exercise training was 3 days a week and 60 min a day, including a 15 min warm-up, 40 min Baduanjin training and 5 min cool down. Baduanjin training was assigned at three community centers (Jinniushan Community Center, Wenquan Community Center and Wufeng Community Center in Fuzhou city) with 15–20 individuals per center. Three professional coaches, who have been engaged in teaching Baduanjin exercise to college students at the Fujian University of Traditional Chinese Medicine (FJTCM) for at least 5 years, were employed to guide participants’ training. The training scheme for the Baduanjin exercise was planned according to the ‘Health Qigong Baduanjin Standard’ enacted by the State Sports General Administration in 2003 (34). The Baduanjin exercise comprises 10 postures (including the preparation and ending postures). A Polar heart rate (HR) monitor (Mio Sport SD) was used to monitor participants’ energy consumption and HR during training.

Participants in the control group were informed to maintain their original lifestyle without any specific exercise intervention.

Additionally, all participants received 6 sessions of health education lectures on nutrition and diet-related knowledge with 30–60 min per session and once every 4 weeks. Furthermore, all participants were asked to record their daily physical activities through a self-designed daily activity questionnaire during the 24-week intervention period.

## Outcome measures

### Cognitive frailty

Global cognitive function was assessed using the Chinese version of the MoCA scale (35). The rating scale covers 8 cognitive domains: visuospatial executive ability, naming, memory, attention, language ability, fluency, abstract thinking, delayed recall, orientation, out of 30 points. The cut-off value was 26 points. The higher the score, the better the global cognitive function. Considering that different educational levels can cause a certain bias, if the total years of education of the research subjects are less than 12 years, the final score would be increased by 1 point (36).

Physical frailty was evaluated by Chinese version EFS scale (37). EFS covers 9 dimensions including cognitive ability, overall health status, functional independence, social support, medication status, nutrition, emotion, control ability, and functional performance. Each dimension is scored 0, 1, and 2 points, with a total score of 17 points. The cut-off value is 6, and the score is negatively correlated with physical function. The higher the score means the more serious the physical frailty (38).

### Oxidative stress markers and inflammatory cytokines

After a 12-h overnight fast, 5 mL whole blood samples were obtained in the morning by an experienced nurse at baseline and 24 weeks after intervention. The 24-week blood samples were collected at least 3 days after the last exercise training to eliminate the short-term effects of exercise.

The MDA assay kit (thiobarbituric acid method), SOD assay kit (xanthine oxidase method), human 8-iso prostaglandin F2α (8-iso-PGF2α) ELISA kit (competitive method), nitric oxide (NO) assay kit (one-step method), T-AOC assay kit (microplate method), and lipid peroxidation (LPO) assay kit were used to measure the content of oxidative stress markers (MDA, SOD, 8-iso-PGF2α, NO, T-AOC and LPO) in serum. All the assay kits used in this study were developed by Nanjing Jiancheng Bioengineering Institute.

The levels of inflammatory cytokines including IL-2, IL-4, IL-6, IL-8, IL-10, TNF-α, IFN-γ and granulocyte macrophage-colony stimulating factor (GM-CSF) in serum were measured using the Bio-Plex Pro Human Cytokine Assays (liquid chip multi-factor detection technology, Bio-Rad Company in the United States) according to the manufacturer’s instructions.

All measurements on serum specimens were conducted by the experienced staff at the medical experiment center of the Academy of Integrative Medicine, Fujian University of Traditional Chinese Medicine.

### Sample size and statistical analysis

The sample size was estimated based on improvements in EFS or MoCA scores. A total of 102 samples were necessary to have 80% power to detect a difference in the target effect EFS or MoCA changes between Baduanjin training and control group after intervention with a maximum loss to follow-up of 20%. This was reported in previously published protocol (32).

The continuous variables were expressed as a mean ± standard deviation (SD) or median and interquartile range (IQR) when appropriate, while the categorical variables were expressed as frequency. The baseline characteristics between groups, including age, sex, education, BMI, BDI and GDS, were examined by the two independent sample t-test/the Mann–Whitney U rank test for continuous variables or the Chi-square test for categorical variables. Between-group differences in cognitive ability and physical frailty, oxidative stress and inflammatory cytokines of two groups at baseline and 24 weeks after intervention were compared by independent t-test or Mann–Whitney U rank test, and the linear mixed model was used to observe the interaction effect of exercise intervention and time-based on the intention-to-treat principle with the missing data handled using the multiple imputation method. To test whether the effect of Baduanjin exercise training on cognitive or physical function was mediated by the inflammatory, antioxidative, or pro-oxidative stress cytokines, we conducted mediation analyses with the significantly changed cytokines as mediators using Hayes’s PROCESS macro (39). All analyses were conducted using SPSS version 26. All tests were two-sided, with *p* < 0.05 indicating statistical significance.

## Results

**Figure 1:**
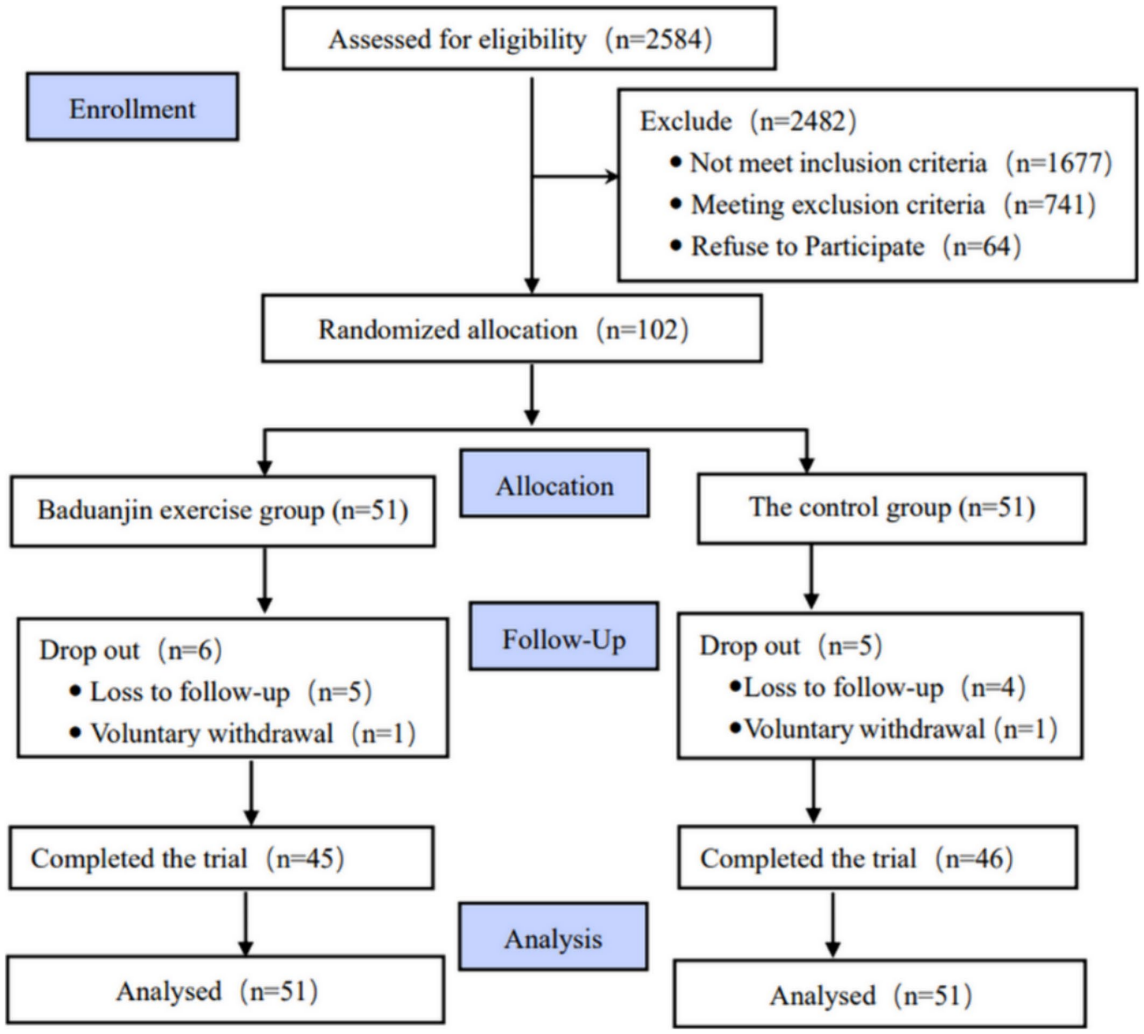
CONSORT flow diagram of the study.

Figure 1 shows the flow diagram of participants’ recruitment, randomization and intervention. Of 2,584 individuals who manifested interest in this study, 2,482 were excluded based on the adopted criteria. 102 eligible older adults performed all baseline assessments and were randomly allocated into the Baduanjin exercise training or control group. During the 24 weeks intervention period, a total of 11 participants (6 in the Baduanjin exercise training group, and 5 in the control group) dropped out of the trial, but no significant difference in dropout rate between two groups was found (*p* > 0.05). Finally, 91 participants completed the intervention. For the Baduanjin exercise training group, some participants did not complete all training plans due to the limitation of bad weather and personal reasons, but the average attendance numbers were up to 57.7 times with the 81.3% adherence rate.

The characteristics of the participants are presented in Table 1. There were no differences in gender, education, BMI, grip strength, GDS and BDI between the groups. The average ages in the Baduanjin group were higher than in the control group (*p* = 0.021).

**Table 1:** Comparison of demographic information and characteristics between two groups (numbers/mean ± SD/median (IQR)).

Baseline characteristic	Baduanjin group (*n* = 51)	Control group (*n* = 51)	t/Z/χ^2^ value	*p* value
Sex (Male/Female)	19/32	20/31	0.042	0.839
Age* (Years)	67.68 ± 5.19	65.35 ± 5.15	−2.305	0.021
Education* (years)	10.90 ± 2.81	10.02 ± 2.90	−1.539	0.124
BMI	23.75 ± 2.33	24.32 ± 3.22	−1.021	0.310
Grip strength	23.31 ± 4.54	23.68 ± 4.21	0.619	0.536
GDS (Level 2 / Level 3)	34/17	36/15	0.182	0.670
BDI *	3.5 (1.75–5)	3.5 (3–5)	0.426	0.670
Physical activity between the two groups (hours)				
Sleep	6.69 ± 1.39	6.66 ± 1.56	−0.167	0.867
Static activity	12.41 ± 2.09	12.75 ± 1.88	1.373	0.170
Low intensity activity	4.77 ± 1.37	4.47 ± 1.60	−1.096	0.273
Medium intensity activity *	1.04 ± 0.14	0.09 ± 0.19	−9.544	<0.001
High intensity activity	0 ± 0.00	0 ± 0.00	NA	NA

*Data did not conform to normal distribution, and independent sample Mann–Whitney U test was used; NA: Not analyzed; GDS, Global Deterioration Scale; BMI, Body Mass Index; BDI, Beck Depression Index.

During the 24-week intervention period, no significant difference was found in the time length for sleep, static activities, low-intensity, and high-intensity activities (*p* > 0.05) between two groups. The time length of moderate-intensity activity in Baduanjin group were significantly higher than in the control group (*p* < 0.001) due to additional Baduanjin exercise training. Baduanjin training was categorized as a moderate-intensity activity according to the percentage of maximum heart rates of practices, and the average 58.86% of maximum heart rates in the Baduanjin training group were monitored (Table 1).

Analysis of outcomes on global cognitive function, physical frailty, blood biomarkers of antioxidant, pro-oxidative and inflammatory factors at baseline and the difference from 24-week intervention to baseline between two groups are presented in Table 2. After 24 weeks intervention, the MoCA scores in the Baduanjin group were averagely increased by 2.51 points (SD = 0.32) and significantly (*p* < 0.001) higher than that in the control group; while EFS scores in Baduanjin group were averagely decreased by 1.9 points (SD = 0.20) and significantly (*p* = 0.012) lower than that in the control group, with a significant interaction effect of treatment by time (*p* < 0.001 for MoCA, and *p* = 0.012 for EFS).

**Table 2:** Comparison of MoCA, EFS, and blood biomarkers of antioxidant, pro-oxidative and inflammatory factors at baseline and difference from 24-week intervention to baseline between two groups [mean ± SD/median (IQR)].

Variables	Control group (*n* = 51)		Baduanjin group (*n* = 51)		*P*_mixed linear model_		
	Baseline	Changes from 24 week to Baseline	Baseline	Changes from 24 week to Baseline	Treatment	Time	Interaction effect
MoCA Score	21.55 ± 3.67	0.34 ± 0.44	22.67 ± 2.83	2.51 ± 0.32^*^	0.001	<0.001	<0.001
EFS Score	5.67 ± 1.16	−1.16 ± 0.23	5.41 ± 0.64	−1.94 ± 0.20^*^	0.002	<0.001	0.012
Antioxidant markers							
NO (μmol/L)	5.6 (2.8–11.1)	−4.75 ± 0.96	5.3 (2.3–8.5)	−3.76 ± 0.94	0.541	<0.001	0.464
T-AOC (mmol/L)	1.24 ± 0.32	−0.00 ± 0.05	1.15 ± 0.31	0.05 ± 0.06	0.155	0.588	0.509
SOD (U/mL)	54.8 ± 18.0	−14.9 ± 3.8	52.51 ± 21.84	10.03 ± 4.73^*^	<0.001	0.419	<0.001
Pro-oxidative markers							
MDA (nmol/mL)	2.30 (1.5–3.5)	1.73 ± 0.99	2.26 (1.65–3.5)	−1.08 ± 0.80^*^	0.016	0.610	0.030
8-iso-PGF2α (ng/L)	203.3 ± 79.3	66.8 ± 15.9	211.6 ± 81.9	−86.6 ± 15.0^*^	<0.001	0.369	<0.001
LPO (μmol/L)	1.69 (0.6–3.6)	−0.01 ± 0.51	2.03 (0.63–4.06)	−0.59 ± 0.51	0.890	0.409	0.422
Inflammatory cytokines							
GM-CSF (pg/mL)	0.69 (0.50–1.87)	0.38 ± 0.31	0.56 (0.50–0.98)	0.74 ± 0.24	0.089	0.005	0.357
IFN-γ (pg/mL)	0.83 (0.61–1.72)	0.10 ± 0.32	0.61 (0.39–0.90)^*^	1.08 ± 0.33^*^	0.880	0.011	0.034
TNF-α (pg/mL)	13.1 (4.9–43.3)	67.8 ± 19.9	8.1 (4.0–14.5)^*^	92.9 ± 19.8	0.758	<0.001	0.374
IL-2 (pg/mL)	2.82 (2.30–5.15)	0.60 ± 0.70	2.82 (2.82–2.82)	2.74 ± 0.75^*^	0.942	0.002	0.040
IL-4 (pg/mL)	0.71 (0.42–1.84)	0.48 ± 0.33	0.40 (0.21–0.75)^*^	1.48 ± 0.35^*^	0.813	<0.001	0.042
IL-6 (pg/mL)	90.3 (28.9–325)	−60.6 ± 79.1	13.2 (1.7–75.1)^*^	29.4 ± 77.1^*^	0.250	0.778	0.417
IL-8 (pg/mL)	1,544 (549–2,550)	−145 ± 442	546 (277–1,308)	919 ± 386^*^	0.164	0.190	0.073
IL-10 (pg/mL)	1.55 (0.48–3.20)	−0.07 ± 0.69	1.01 (0.48–2.36)	0.41 ± 0.58	0.676	0.709	0.599

*<0.05 compared to the control group. MoCA, Montreal Cognitive Assessment; EFS, Edmonton Frail Scale; NO, Nitric Oxide; T-AOC, Total Antioxidant Capacity; SOD, Superoxide Dismutase; MDA, Malondialdehyde; 8-iso-PGF2α, 8-iso-prostaglandin F2α; LPO, Lipid Peroxidation; GM-CSF, Granulocyte Macrophage-colony Stimulating Factor; IFN-γ, Interferon-gamma; TNF-α, Tumor Necrosis Factor-alpha; IL, Interleukin.

Serum antioxidant marker SOD levels were decreased by 14.9 U/mL (SD = 3.8) in Baduanjin training group but increased by 10.03 U/mL (SD = 4.74) in the control group after 24 weeks intervention, and the comparison of difference from baseline to 24-week intervention was significant (*p* < 0.05) with a significant treatment effect (*p* < 0.001) and interaction effect of treatment by time (*p* < 0.001).

Serum pro-oxidative markers MDA and 8-iso-PGF2α levels were increased in the Baduanjin training group but decreased in the control group after 24 weeks intervention, and comparison of differences between two groups was significant (*p* < 0.05) with a significant treatment effect (*p* = 0.016 for MDA and *p* < 0.001 for 8-iso-PGF2α) and interaction effect of treatment by time (*p* = 0.03 for MDA and *p* < 0.001 for 8-iso-PGF2α).

For serum inflammatory cytokines, the IFN-γ, IL-2 and IL-4 levels in Baduanjin training group were significantly (all *p* < 0.05) lower than them in the control group at baseline, but the increased values were significantly (all *p* < 0.05) higher than them in control group after 24 weeks intervention with the significant treatment by time interaction effect (*p* = 0.034, *p* = 0.04, and *p* = 0.042, respectively). While no significant treatment effect (*p* = 0.880, *p* = 0.942, and *p* = 0.813, respectively) indicates the comparison of three cytokines levels between two groups were not significantly different at the 24-week intervention time point. The changes of IL-6 and IL-8 from 24-week to baseline in the Baduanjin exercise group were significantly higher than them in the control group but no treatment and interaction effect of treatment by time was found.

**Figure 2:**
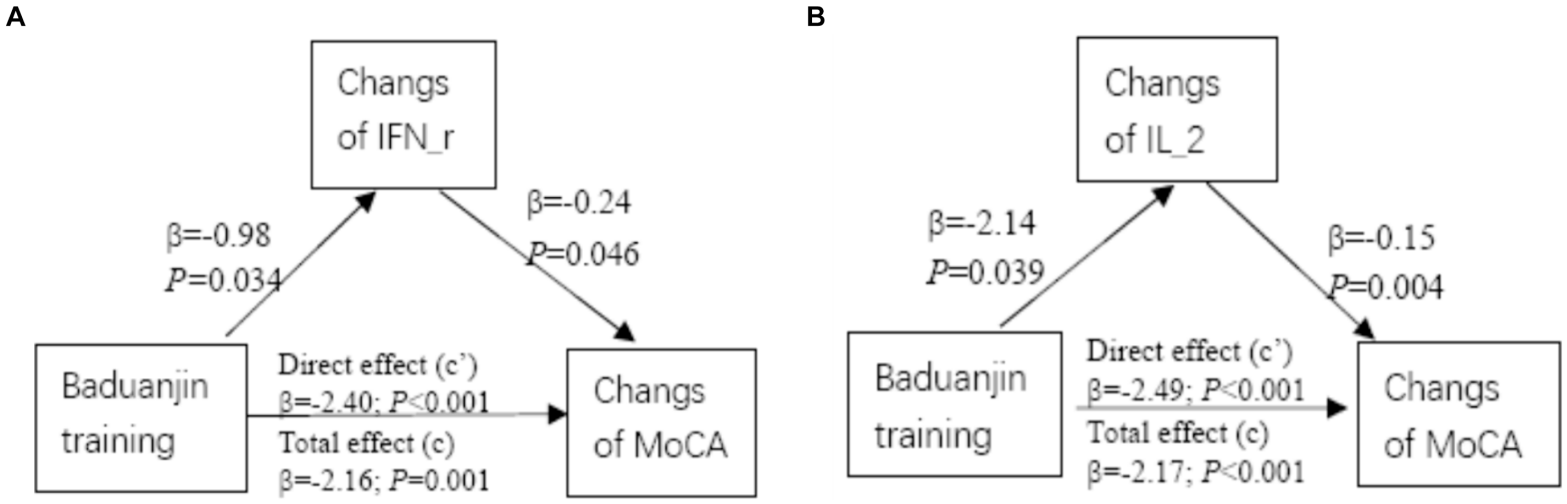
Mediation analysis. Diagram representing the mediation model in which Baduanjin exercise training mediates serum IFN-r or IL-2-induced increases in MoCA scores. Unstandardized coefficients are depicted in each respective pathway on the graph.

To further investigate the effect of Baduanjin exercise training on cognitive or physical function was mediated by the inflammatory or anti−/pro-oxidative markers, the mediation analysis was performed using Baduanjin training as the predictive variable, the changes of MoCA/EFS scores as the dependent variables, and the significantly changed inflammatory and anti−/pro-oxidative markers (SOD, MDA, 8-iso-PGF2a, IFN-r, IL-2, and IL-4) respectively as mediation variables. The results showed a significant indirect effect of Baduanjin training on the improvement of cognitive function (MoCA scores increases) via their increase of serum INF-r (*β* = 0.23, 95%:0.01 ~ 0.63) or IL-2 (*β* = 0.32, 95% CI: 0.02 ~ 0.72) levels. The graphical depictions of the model are seen in Figure 2, along with the statistics measuring the significance of each predictive pathway.

## Discussion

This study was conducted to investigate the effect of 24-week Baduanjin exercise intervention on CF outcomes, oxidative stress markers, and inflammatory cytokines for community-dwelling older adults with CF. The results showed that 24 weeks of regular Baduanjin exercise training could significantly increase the MoCA scores and decrease the EFS score, as well as increase the activity of antioxidant marker SOD, decrease pro-oxidative markers MDA and 8-iso-PGF2α levels, and effectively regulate the levels of inflammatory cytokines IFN-γ, IL-2, and IL-4 level, compared to no specific exercise intervention control. Furthermore, we also found a significant mediation effect of Baduanjin exercise intervention on the improvement of cognitive function mediated by serum INF-r and IL-2. The findings suggest regular Baduanjin training could be beneficial to improve CF and modulate the oxidative stress or inflammation status of the community-dwelling older population, while part inflammatory cytokines might play a mediating role between Baduanjin training and the improvement of cognitive function.

The MoCA scale is currently the most used scale for determining cognitive impairment, showing high sensitivity in detecting early cognitive changes in various cognitive domains, with high sample heterogeneity and detection rate (40, 41). While the EFS scale has been widely used in the clinical frail assessment of the older adults with good structural validity, reliability, and internal consistency (42, 43). Therefore, the combinational assessment of cognitive ability and physical frailty can comprehensively express the CF status of an older person (44, 45). Baduanjin is one of the traditional Chinese qigong exercises, with the characteristics of symmetrical body postures and movements, allowing the interaction among body movement, meditation and breath to coexist harmoniously. Modern researches showed that Baduanjin exercise could regulate cognition-related brain functions and structures, and improve attention, executive control and memory of older adults with/without cognitive impairment (46, 47). On the other hand, Baduanjin exercises also emphasize the dynamic coordination of the upper and lower limbs of the practitioners, taking the lumbar spine as the axis to maintain a stable gravity. Therefore, it could increase the gait, balance ability, and strength of the upper and lower limbs to improve physical function (48–50). The current study found that after 24 weeks of training, the average MoCA scores in the Baduanjin exercise training group were increased by 2.51 (SD = 0.32) points, while average EFS scores were decreased by 1.94 (SD = 0.20) points, and had a significant difference compared to the control group, with the significant interaction effect of treatment by time. Those findings are consistent with the previous research results, indicating that a 24-week Baduanjin exercise training can improve the cognitive ability and physical frailty of community-dwelling older adults with CF.

Although the causes of CF are complex and multidimensional, increasing studies indicate oxidative stress and inflammation play an important role in the development of CF in older adults (9, 51–53). While regular exercise with appropriate intensity can help activate salient cell adaptive properties to achieve a state of oxidative eustress (54). For example, regular moderate-intensity exercise could protect from oxidative damage by enhancing the expression of antioxidant key enzymes SOD, inhibiting pro-oxidative species such as NO, MDA and 8-iso-PGF2α (55–58). Our study found that 24-week Baduanjin exercise training could significantly increase the serum antioxidant stress marker SOD level, as well as decrease pro-oxidative stress MDA and 8-iso-PGF2α levels of older adults with CF compared to the controls, with a significant interaction effect of treatment by time. The findings are in line with the results of our previous systematic review, in which we found regular aerobic exercises had a positive effect on the oxidative stress levels of older adults by reducing pro-oxidant markers MDA and LPO levels and increasing antioxidant markers SOD and TAC levels (59), indicating that 24 weeks Baduanjin training might be helpful to improve the oxidation-antioxidant balance in the older adults with CF by modulating serum levels of antioxidative and pro-oxidative stress markers.

Regular physical activities or exercises are effective in regulating systemic inflammation of the older adults, and the dominant opinions thought they should be beneficial to reduce the levels of inflammatory cytokines in older adults (60). However, there is no consensus on the modulating effect of different exercise modes (i.e., aerobic, resistance, or mind–body exercise) for different inflammatory cytokines (60). One major cause should be related to different exercise intensities and oxygen consumption for different exercise models (61, 62). Another critical cause should result from the dual function (pro−/anti-inflammatory) of some inflammatory cytokines. For example, the IL-2, IL-6, IL-8, IL-10, IFN-γ, and TNF-α possess pro-or anti-inflammatory roles but usually are against pro-inflammatory by regulating pro-inflammatory cytokine responses (63). In the present study, 24-week Baduanjin training showed a significantly increased effect for serum IFN-γ, IL-2, IL-4 and IL-8 levels with a significant interaction of treatment by time for IFN-γ, IL-2 and IL-4. In addition, we also found an obvious mediation effect of the 24-week Baduanjin exercise intervention on the improvement of cognitive ability mediated by regulating the inflammatory cytokines IFN-γ and IL-2 levels. However, the results of this trial are not a consensus with the current majority of studies. A recent exploratory study conducted in institutionalized older adults also showed an 8-week multimodal exercise training (including aerobic, balance/flexibility, and perception/cognition composition) could increase the plasma IL-2 and IFN-αlevels (64). Another single-arm trial reported a 3-month Yoga and meditation intervention could increase plasma IFN-γ, TNF-α, IL-6 and IL-8 levels in middle-aged adults (65). These previous studies provided limited support for current findings, possibly indicating potentially different pathways of mind–body exercise on the inflammatory response.

### Limitations

There are some limitations worth noting in this study. Firstly, the timepoint that participants entered in the trial was not the same, and the serum samples were collected at different timepoint. To save manpower and budget, those serum samples were experimented with at the same time. Although these serum samples were detected within their valid testing period, potential bias is not ignored due to some serum samples being stored too long causing obvious variability of experimental data in some variables, especially those inflammatory cytokines. Future studies should timely test those clinical serum specimens to avoid variability in certain serum variables. Secondly, while a positive effect of regular Baduanjin exercise training improving CF and modulating some oxidative stress or inflammatory cytokines was observed, more work might be required to elucidate the underlying mechanism to explain the causality. Thirdly, only no specific exercise intervention group was designed as the control in this study, therefore we did not compare the intervention effect between Baduanjin exercise and other conventional exercise types (e.g., jogging and swimming). In future studies, different exercise types should be added to compare the advantages and characteristics of the Baduanjin intervention. Fourth, in the trial randomization, we did not use a stratified design, so there was a significant difference in the age of the two groups, which may lead to bias in the research results. Finally, although those older adults suffering from major chronic diseases were excluded from the study, and reduced selection bias to the greatest extent through a randomized controlled design, the occurrence of some acute and transient inflammation during the intervention may significantly increase cytokine expression given the susceptibility of the older adults, leading to the variability of results. Future studies with more rigorous and more samples are necessary to reduce this confounding effect.

## Conclusion

A 24-week regular Baduanjin exercise training could improve the physical frailty and cognitive function of the community-dwelling older adults with CF, seems to modulate oxidative stress and inflammatory processes by a reducing in circulating pro-oxidative MDA and 8-iso-PGF2α levels and an increasing of anti-oxidative SOD levels, as well as impacting inflammatory cytokines IFN-r, IL-2 and IL-4 levels. But the mechanism that Baduanjin exercise improves CF of older adults mediated by modulating oxidative stress and inflammatory processes should be cautious to be explained through a mediation effect that Baduanjin training increased cognitive ability mediated by an increase of circulating IL-4 levels was found in this study. Thus, this mechanism should be confirmed in more studies with a larger sample size and a more rigorous study design in the future.

## Data availability statement

The original contributions presented in the study are included in the article/supplementary material, further inquiries can be directed to the corresponding author.

## Ethics statement

This study was approved by the Ethics Committee of the Second Affiliated Hospital of Fujian University of Traditional Chinese Medicine (No. 2018-KL015). The studies were conducted in accordance with the local legislation and institutional requirements. The participants provided their written informed consent to participate in this study.

## Author contributions

YY: Investigation, Project administration, Writing – original draft. MW: Investigation, Writing – original draft. HL: Supervision, Writing – review & editing. RX: Methodology, Investigation, Writing – review & editing. JH: Writing – original draft. PQ: Investigation, Writing – review & editing. GZ: Conceptualization. Funding acquisition, Project administration, Writing – review & editing.

## Footnotes

^1^ http://www.chictr.org.cn/index.aspx
